# Glucagon-Like Peptide-1 (GLP-1) Receptor Agonists in Obese Patients Without Diabetes: A Systematic Review and Meta-Analysis

**DOI:** 10.7759/cureus.95938

**Published:** 2025-11-02

**Authors:** Roaa Mohamed Ali Elbashir, Azza Elbashir, Robert Urimuke Basake, Amna Zakaria Ahmed Mohieldin, Najla I A Elhaj, Fatima Ebrahim Mohamed Ebrahim, Waad Gase Ahmed, Ola Abdelrahim Mohamed Mahgoub

**Affiliations:** 1 General Practice, Barnsley Hospital NHS Foundation Trust, Barnsley, GBR; 2 Department of Medicine, Barnsley District General Hospital, Barnsley, GBR; 3 Department of Acute Medicine, Aberdeen Royal Infirmary, Aberdeen, GBR; 4 Department of Medicine, Ahfad University for Women, Omdurman, SDN; 5 Pediatric Department, Royal Edinburgh Hospital, Edinburgh, GBR; 6 Emergency Medicine, SABA Clinic, Jeddah, SAU; 7 Department of Medicine, University of Khartoum, Khartoum, SDN

**Keywords:** diabetes, diabetes type 2, glp-1 receptor agonists, type 1 diabetes mellitus, weight reduction

## Abstract

Glucagon-like peptide-1 (GLP-1) receptor agonists (RAs) treat overweight or obesity with or without diabetes. This review aims to evaluate the effects of GLP-1 RAs on weight and cardiometabolic measures. This systematic review and meta-analysis of randomized trials from PubMed, Scopus, Web of Science, and Embase followed the Preferred Reporting Items for Systematic Reviews and Meta-Analyses (PRISMA) guidelines. Two reviewers extracted the data and assessed the quality with the Cochrane Risk of Bias 2 (RoB 2) tool. Thirteen randomized controlled trials were included. We concluded that GLP-1 RAs reduced mean percentage body weight by -12.79% (95% CI: -15.12 to -10.46), BMI by -4.80 kg/m² (95% CI: -6.24 to -3.36), waist circumference by -9.78 cm (95% CI: -11.47 to -8.09), systolic blood pressure (BP) by -6.32 mmHg (95% CI: -7.92 to -4.72), and diastolic BP by -1.95 mmHg (95% CI: -3.21 to -0.69). Risk ratios for ≥5%, ≥10%, ≥15%, and ≥20% weight loss were 2.98, 5.56, 9.50, and 15.00, respectively. Tirzepatide showed greater reductions than semaglutide. GLP-1 RAs, particularly tirzepatide, achieve substantial weight loss and improve cardiometabolic risk factors.

## Introduction and background

Glucagon-like peptide-1 (GLP-1) is an incretin hormone secreted by the intestine after food intake, where it enhances glucose-dependent insulin release from pancreatic β-cells, as mentioned in a study done by Vosoughi et al. (2022) [1]. It also reduces glucagon secretion from α-cells, thereby lowering hepatic glucose production in a glucose-dependent manner. This suppression of glucagon may further be modulated through a paracrine effect by somatostatin released from neighboring δ-cells [2]. GLP-1 receptor agonists (RAs) mimic these physiological actions, resulting in reductions of glycosylated hemoglobin (HbA1c) by approximately 1.0-1.5% and lowering postprandial glucose concentrations [2].

Excess body weight is a well-established risk factor for conditions such as cardiovascular disease, type 2 diabetes, chronic kidney disease, and certain cancers. In 2016, global estimates indicated that 1.9 billion adults were overweight, of whom more than 650 million were classified as obese. While lifestyle interventions remain central to obesity management, pharmacotherapy is an important adjunct for individuals with overweight or obesity who require additional support. Comparative reviews and meta-analyses of available drugs consistently identify GLP-1 RAs as among the most effective treatments for weight reduction in adults with overweight or obesity. These agents influence both central and peripheral pathways controlling appetite, food intake, and glucose regulation [1].

Beyond their role in patients with diabetes, accumulating evidence shows that GLP-1 RAs also facilitate weight loss in individuals with overweight or obesity who do not have diabetes. Since 2019, semaglutide has been increasingly studied in large-scale randomized controlled trials (RCTs) involving non-diabetic populations, consistently showing significant weight reduction [3].

The therapeutic landscape of GLP-1 RAs has progressed rapidly, with agents initially available only via subcutaneous injection now also formulated for oral use. Newer oral GLP-1 RAs include semaglutide and orforglipron. Updated evidence syntheses are needed to integrate findings from these recent large RCTs, comparing both individual and pooled effects of established and novel agents, as well as contrasting injectable and oral formulations in people with overweight or obesity. Importantly, most prior reviews have focused on populations with diabetes, leaving knowledge gaps regarding the relative benefits of GLP-1 RAs in non-diabetic patients with obesity. Comparative evaluation of outcomes in those with and without diabetes is therefore warranted [4].

## Review

Methodology

This review was conducted in accordance with the Preferred Reporting Items for Systematic Reviews and Meta-Analyses (PRISMA) guidelines and applied the PICOS (population, intervention, comparison, outcomes, and study design) framework to structure both the development and reporting process. The article has been submitted to the International Prospective Register of Systematic Reviews (PROSPERO; ID: CRD420251176587).

Eligibility Criteria

The methodological rigor of the included RCTs was evaluated using the Cochrane Risk of Bias 2 (RoB 2) tool. Two reviewers independently assessed each study across five domains: (1) adequacy of the randomization process; (2) adherence to intended interventions; (3) completeness of outcome data; (4) accuracy of outcome measurement; and (5) selective reporting of results. Each domain was rated as low risk, some concerns, or high risk based on signaling questions. An overall judgment for each study was then determined, with disagreements resolved through consensus discussions.

Search Strategy

A comprehensive literature search was performed in PubMed, Scopus, Web of Science (WOS), and Embase. Only articles published in English were considered. The search employed Boolean operators and free-text terms aligned with each PICOS element to maximize coverage of relevant studies. Core search terms included the following: “GLP-1 receptor agonist”, “glucagon-like peptide-1 receptor agonist”, “semaglutide”, “liraglutide”, “obesity”, and “diabetes” or “diabetic”. Search terms were applied to titles, abstracts, and subject headings, and no restrictions on publication date were imposed. Retrieved records were imported into Rayyan (Cambridge, MA), a web-based tool, for de-duplication and screening.

Study Screening

**Figure 1:**
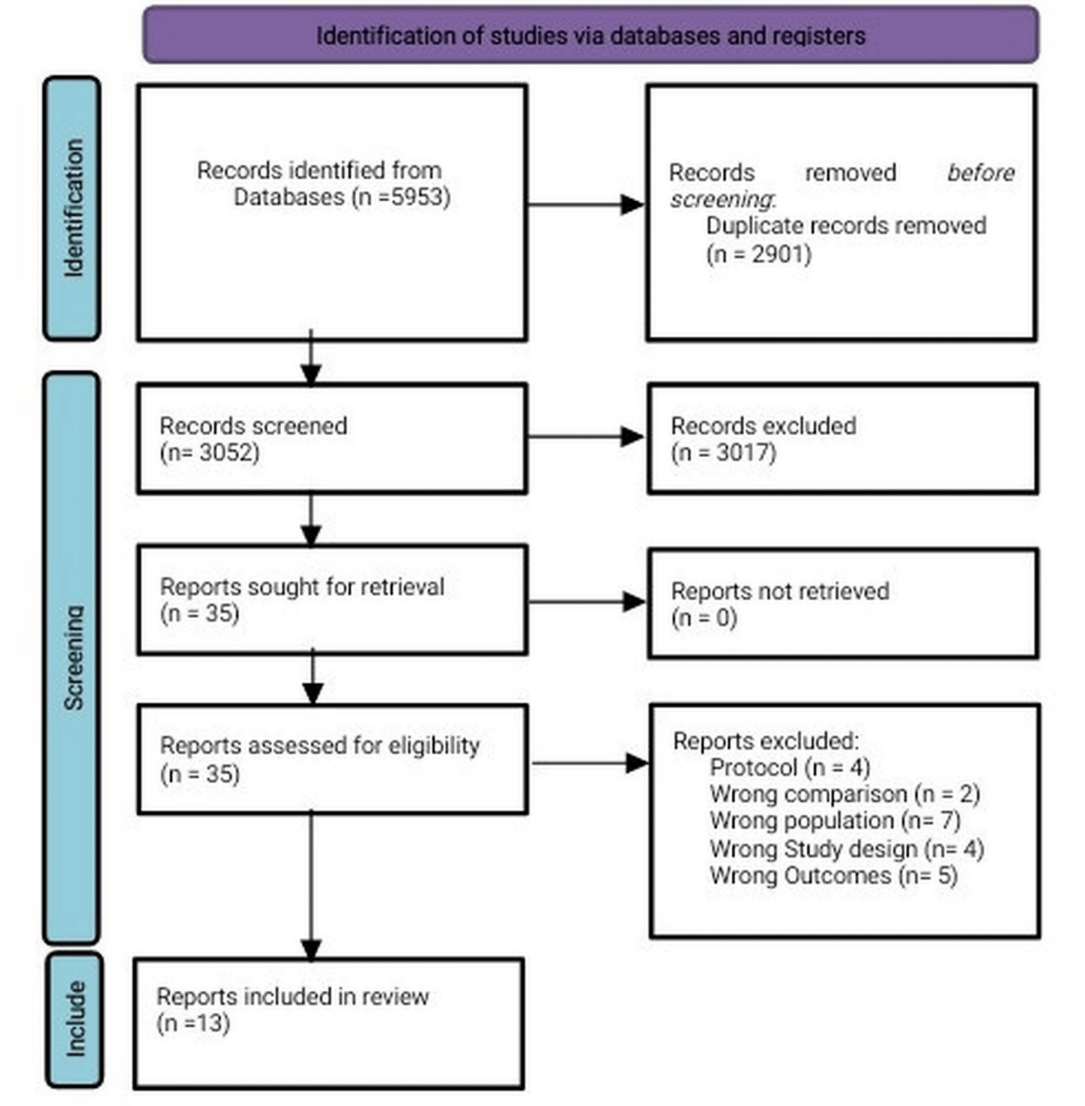
Studies included and excluded in primary and secondary screening. Rayyan (Rayyan, Cambridge, MA) is used for the screening.

After automatic removal of duplicates in Rayyan, two reviewers independently screened titles and abstracts for relevance. Studies meeting criteria were subjected to full-text review. Discrepancies between reviewers were resolved through discussion and, if necessary, consultation with a third reviewer. All screening decisions and reasons for exclusion were documented, and the overall selection process was summarized in a PRISMA flow diagram (Figure 1).

Data Extraction

Data extraction was performed independently by two authors using a standardized form, with subsequent cross-checking for accuracy. Extracted information included trial identifiers, design, registry number, study location, randomization procedures, interventions and comparators, sample size, and follow-up duration. All studies included in the article are summarized in Table 1.

**Table 1:** Details of all the studies included in the systematic review. NICE: National Institute for Health and Care Excellence; N/V: no value; HbA1c: glycosylated hemoglobin; FPG: fasting plasma glucose; OSA: obstructive sleep apnea; CVD: cardiovascular disease; DM: diabetes mellitus; SC: subcutaneous; JASSO: Japan Society for the Study of Obesity; MASLD: metabolic dysfunction-associated steatotic liver disease; GLP-1 RAs: glucagon-like peptide-1 receptor agonists.

Author	Study ID	Study design	Registry number	Country	Randomization method	Recruitment period	Intervention		Control		No. of participants			Follow-up	Main inclusion criteria
							Type	Description	Type	Description	Intervention	Control	Total		Into small points (paraphrased)
Apperloo et al. (2025) [5]	N/V	Randomized, double-blind, placebo-controlled, parallel-group, phase 3 clinical trial	NCT04889183	The Netherlands, Germany, Spain, Canada (Multicenter)	Computer-generated pseudo-random number generator (1:1 ratio)	March 2022 to November 2023	Semaglutide	Subcutaneous semaglutide, once weekly, up-titrated to a 2.4 mg dose	Placebo	Placebo	51	50	101	24-week treatment period, plus a 4-week post-treatment washout assessment	• Age ≥ 18 years. • BMI ≥ 27 kg/m². • Chronic kidney disease (CKD) without diabetes. • Urine albumin-to-creatinine ratio (UACR) ≥ 30 and ≤3,500 mg/g. • Estimated glomerular filtration rate (eGFR) ≥ 25 ml/min/1.73 m². • Stable renal function (eGFR change < 30% in 3 months prior)
Barbara et al. (2024) [6]	STEP 10	Randomized, double-blind, placebo-controlled, parallel-group, phase 3 trial	NCT05040971	Canada, Denmark, Finland, Spain, UK	2:1 randomization via interactive web-response system	16 September 2021 to 29 December 2021	Semaglutide	Once-weekly subcutaneous semaglutide 2.4 mg + diet/physical activity counseling	Placebo	Placebo + diet/physical activity counseling	138	69	207	52 weeks (primary endpoint) + 28-week off-treatment period (total 80 weeks)	• Adults ≥18 years. • BMI ≥30 kg/m² (obesity) - Prediabetes (UK NICE criteria): • HbA1c of 6·0–6·4% (42–47 mmol/mol) • OR FPG: 5.5-6.9 mmol/L - No diabetes history
Garvey et al. (2022) [7]	STEP 5	Phase 3, randomized, double-blind, placebo-controlled, multinational	NCT03693430	Canada, Italy, Hungary, Spain, United States (5 countries)	1:1 central randomization via interactive web-response system (IWRS); fixed-size blocking schema	5 October 2018 to 1 February 2019	Semaglutide	Once-weekly subcutaneous semaglutide 2.4 mg + behavioral intervention (diet/exercise counseling)	Placebo	Placebo + behavioral intervention	152	152	304	104 weeks (2 years)	• Adults (≥18 years). • BMI ≥30 kg/m² (obesity) OR BMI ≥27 kg/m² (overweight) + ≥1 weight-related comorbidity (hypertension, dyslipidemia, OSA, CVD). • History of unsuccessful dietary weight loss - without diabetes (HbA1c <6.5%)
Jastreboff et al. (2022) [8]	SURMOUNT-1	Phase 3, double-blind, randomized, placebo-controlled trial	NCT04184622	119 sites across 9 countries	1:1:1:1 ratio, stratified by country, sex, and prediabetes status	December 2019 to April 2022	Tirzepatide	Once-weekly doses (5 mg, 10 mg, or 15 mg) + lifestyle intervention. Dose escalated over 20 weeks (starting at 2.5 mg, increased by 2.5 mg every 4 weeks)	Placebo	Placebo + lifestyle intervention	5 mg: 630; 10 mg: 636; 15 mg: 630	643	2,539	72-week primary treatment period (+ 4-week safety follow-up)	• Adults ≥18 years. • BMI ≥30 or BMI ≥27 + ≥1 weight-related complication (e.g., hypertension, CVD). • ≥1 prior unsuccessful dietary weight loss attempt. • No history of DM
Kadowaki et al. (2025) [9]	SURMOUNT-J	Multicenter, randomized, double-blind, placebo-controlled phase 3 trial	NCT04844918	Japan (18 research centers)	1:1:1 via computer-generated sequence; stratified by comorbidities and sex	May 10, 2021 to June 24, 2023	Tirzepatide	Once-weekly SC tirzepatide (10 mg or 15 mg) + lifestyle modifications	Placebo	Placebo + lifestyle modifications	10 mg: 73; 15 mg: 77	75	225	72-week treatment + 4-week safety follow-up	• Japanese adults (age ≥20). • Obesity disease per JASSO criteria: BMI ≥27 kg/m² + ≥2 comorbidities (e.g., hyperlipidemia, impaired glucose tolerance, MASLD) or BMI ≥35 kg/m² + ≥1 comorbidity. • Excluded type 2 diabetes
Knop et al. (2023) [10]	OASIS 1	Randomized, double-blind, placebo-controlled, phase 3 superiority trial	NCT05035095	Multinational (50 centers across Asia, Europe, North America)	Interactive web-response system (1:1), block size of 4	September 13 to November 22, 2021	Semaglutide	50 mg once daily + lifestyle intervention. Dose escalation: 3 mg → 7 mg → 14 mg → 25 mg → 50 mg over 16 weeks	Placebo	Placebo + lifestyle intervention	334	333	667	68 weeks treatment + 7 weeks off-treatment follow-up (75 weeks total)	• Age: ≥18 years (≥20 years in Japan). • BMI: ≥30 kg/m² or ≥27 kg/m² with ≥1 weight-related complication (hypertension, dyslipidemia, obstructive sleep apnea, cardiovascular disease). • Weight loss effort: At least one prior self-reported dietary weight loss attempt. • Diabetes status: No history of type 1 or type 2 diabetes. Confirmed at screening (HbA1c <6.5%/<48 mmol/mol, medical records, medication, glucose variables).
Li et al. (2023) [11]	N/V	Multicenter, randomized, double-blind, placebo-controlled, phase 2 trial	NCT04799327	China	Interactive web response system; 1:1:1:1 final ratio	29 March 2021 to 4 March 2022	Noiiglutide	Subcutaneous noiiglutide (0.12, 0.24, or 0.36 mg/day)	Placebo	Placebo	0.12 mg: 64; 0.24 mg: 65; 0.36 mg: 63	62	254	24-week treatment + 1-week follow-up	1. Aged 18-65 years. 2. BMI between 28.0 and 40.0 kg/m². 3. History of diet and exercise management for ≥3 months before screening. 4. <5% change in body weight in the previous 3 months. 5. Without diabetes.
Lim et al. (2025) [12]	STEP 11	Randomized, double-blind, placebo-controlled, phase 3b trial	NCT04998136	South Korea and Thailand	2:1 ratio via computer-generated sequence and block randomization (using 4G Clinical software)	August 15, 2022, to November 20, 2023	Semaglutide	Once-weekly subcutaneous semaglutide (2.4 mg) + lifestyle intervention (reduced-calorie diet, increased physical activity)	Placebo	Placebo + lifestyle intervention	101	49	150	44 weeks of treatment + 5-week follow-up	• Adults of Asian descent. • Age: ≥18 years in Thailand; ≥19 years in South Korea. • Obesity defined as BMI ≥25 kg/m². • Without diabetes. • History of at least one self-reported unsuccessful weight-loss attempt
Lincoff et al. (2023) [13]	SELECT	Multicenter, double-blind, randomized, placebo-controlled, event-driven superiority trial	NCT03574597	41 countries (804 sites)	1:1 via centralized system (no stratification)	October 2018–March 2021	Semaglutide	Once-weekly subcutaneous semaglutide 2.4 mg (dose-escalated from 0.24 mg over 16 weeks)	Placebo	Placebo	8803	8801	17604	Mean: 39.8 months (SD: 9.4)	1. Age ≥ 45 years. 2. BMI ≥ 27 kg/m². 3. Pre-existing CVD. 4. No diabetes (HbA1c <6.5%). 5. No glucose-lowering drugs or GLP-1 RAs within 90 days.
Wadden et al. (2021) [14]	STEP 3	Randomized, double-blind, parallel-group, phase 3a	NCT03611582	USA	2:1 via interactive web-response system (block size of 9)	August 2018–November 2018	Semaglutide	Once-weekly subcutaneous semaglutide 2.4 mg + initial 8-week low-calorie diet (1000-1200 kcal/day) + 68-week intensive behavioral therapy (30 counseling visits)	Placebo	Placebo injection + identical low-calorie diet and behavioral therapy	407	204	611	68 weeks (+7 weeks safety follow-up)	1. Adults (≥18 years). 2. Overweight (BMI ≥27 + ≥1 comorbidity) or obesity (BMI ≥30). 3. Prior unsuccessful weight-loss attempts. 4. No diabetes (HbA1c <6.5%). 5. Stable weight (±5 kg in 90 days pre-screening.
Wadden et al. 2023 [15]	SURMOUNT-3	Randomized, double-blind, placebo-controlled, parallel-group, multicenter phase 3 trial	NCT04657016	USA, Argentina, Brazil	Computer-generated random sequence via interactive web-response system (1:1 ratio), stratified by country, sex, and % weight loss during lead-in period	12 April 2021 to 3 September 2021	Tirzepatide	Tirzepatide maximum tolerated dose (10 or 15 mg) once-weekly subcutaneous injection for 72 weeks. Starting dose: 2.5 mg with 2.5 mg increments every 4 weeks.	Placebo	Placebo	287	292	579	72 weeks (primary endpoint)	• Adults (≥18 years) with BMI ≥30 kg/m² OR BMI ≥27 kg/m² with ≥1 weight-related complication. • Achieved ≥5% weight reduction during the 12-week intensive lifestyle intervention lead-in period. • History of ≥1 unsuccessful dietary weight loss attempt. • Willing to self-inject study drug and comply with protocols. • Stable body weight (<5 kg change in past 3 months)
Wilding et al. (2021) [16]	STEP 1	Randomized, double-blind, placebo-controlled trial	NCT03548935	16 countries across Asia, Europe, North America, and South America	2:1 ratio via an interactive web-based response system	June 2018 to November 2018	Semaglutide	Once-weekly subcutaneous semaglutide (2.4 mg) + lifestyle intervention	Placebo	Placebo + lifestyle intervention	1306	655	1961	68 weeks of treatment + 7-week follow-off period	• Adults ≥18 years old. • BMI ≥30 or BMI ≥27 with at least one weight-related condition, such as hypertension or dyslipidemia. • History of unsuccessful dietary weight loss efforts. • Without diabetes
Zhao et al. (2024) [17]	SURMOUNT-CN	Randomized, double-blind, placebo-controlled, phase 3 trial	NCT05024032	China	Computer-generated random sequence via interactive web response system (1:1:1 ratio), stratified by sex and presence of comorbidities	September 2021 - December 2022	Tirzepatide	Once-weekly subcutaneous tirzepatide (10 mg or 15 mg) + lifestyle intervention	Placebo	Placebo + lifestyle intervention	141	69	210	56 weeks total (52-week treatment + 4-week safety follow-up)	• Chinese adults (≥18 years). • BMI ≥28 or BMI ≥24 with ≥1 weight-related comorbidity (hypertension, dyslipidemia, etc.). • ≥1 unsuccessful dietary weight loss attempt. • Without diabetes

Quality Assessment

The overall methodological quality of the included studies was rated as high, with most judged to be at low risk of bias. Domains such as randomization (D1), outcome measurement (D4), and selective reporting (D5) consistently showed low risk, reflecting sound study design, reliable outcome evaluation, and transparent reporting practices. Minor concerns were noted in two studies regarding deviations from intended interventions (D2) and in three studies related to missing outcome data (D3). However, these issues were limited in scope and did not raise any trial to a high-risk rating.

To assess potential publication bias, alternative funnel plots specifically designed for meta-analyses of proportions were employed, as traditional funnel plots may yield misleading interpretations in this context due to the inherent asymmetry of proportion data. The visual inspection of these plots revealed no substantial asymmetry, suggesting an absence of small-study effects. This finding was further supported by Begg’s test, which indicated no statistically significant evidence of publication bias for either full vaccination coverage (z = -0.70, p = 0.48) or measles-containing vaccine coverage (z = -0.27, p = 0.79). These results collectively suggest that the pooled estimates are unlikely to have been influenced by the selective publication of studies.

**Figure 2:**
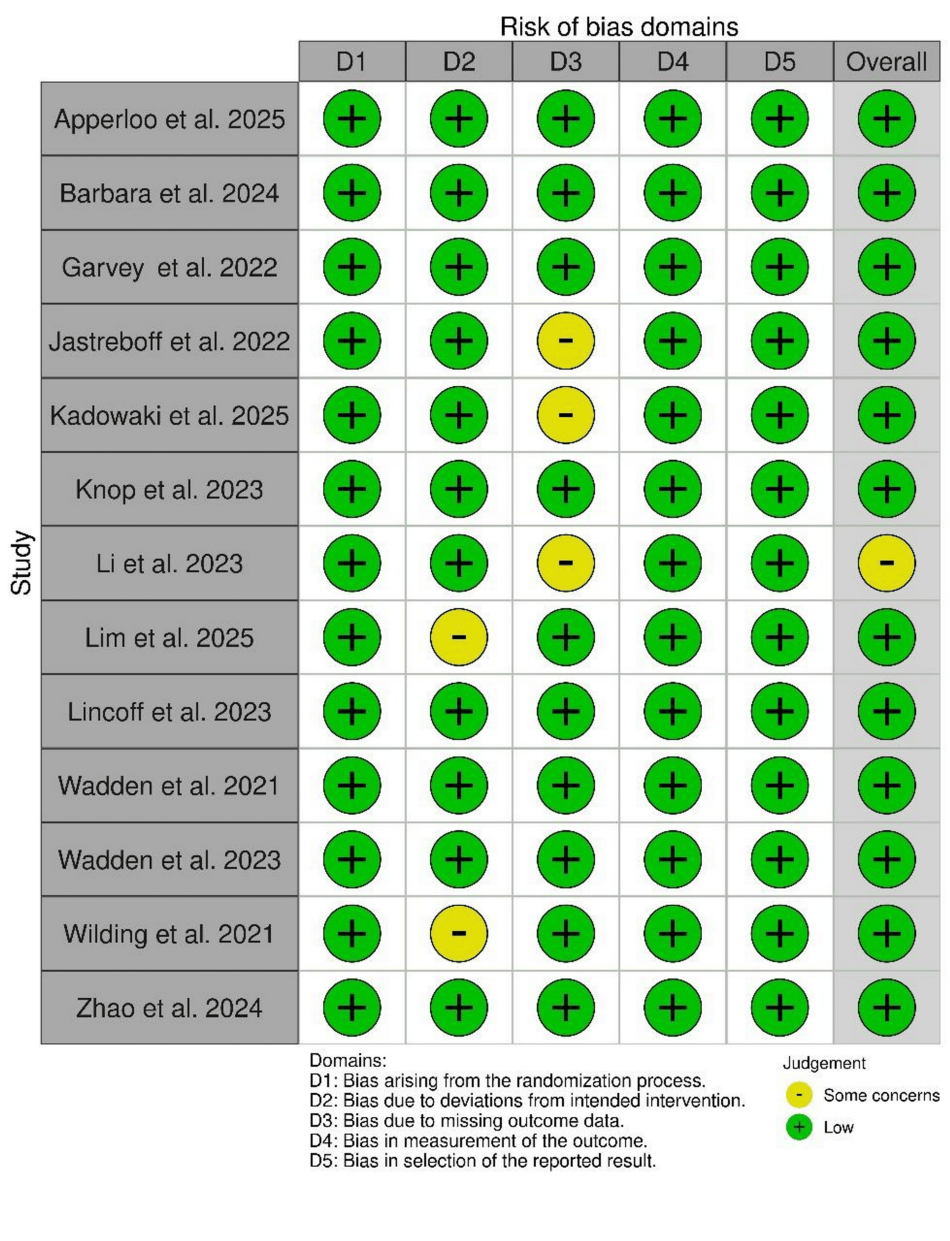
Quality assessment of the studies and the five domains. Apperloo et al. (2025) [5], Barbara et al. (2024) [6], Garvey et al. (2022) [7], Jastreboff et al. (2022) [8], Kadowaki et al. (2025) [9], Knop et al. (2023) [10], Li et al. (2024) [11], Lim et al. (2025) [12], Lincoff et al. (2023) [13], Wadden et al. (2021) [14], Wadden et al. (2023) [15], Wilding et al. (2021) [16], Zhao et al. (2024) [17].

Overall, the evidence base was considered robust, and the limited concerns identified were unlikely to materially affect the validity of the findings (Figure 2).

Statistical Analysis

All statistical analyses were performed using R statistical software, version 4.4.2 (R Foundation for Statistical Computing, Vienna, Austria), with the meta package. For continuous outcomes, the treatment effect was estimated using the mean difference (MD) along with 95% confidence intervals (CIs). For dichotomous outcomes, the treatment effect was estimated using the risk ratio (RR) with 95% CIs.

A random-effects model was applied to all primary meta-analyses to account for anticipated clinical and methodological heterogeneity among the included studies. The magnitude of statistical heterogeneity was quantified using the I² statistic, which was interpreted as follows: 25% (low), 50% (moderate), and 75% (high).

To assess the robustness of the pooled results, a leave-one-out sensitivity analysis was conducted by iteratively removing each individual study and recalculating the summary estimate. Pre-specified subgroup analyses were performed to explore potential sources of heterogeneity, with a primary focus on the specific GLP-1 RA used (semaglutide, tirzepatide, noiiglutide). The statistical significance of differences between subgroups was evaluated.

Potential for publication bias was assessed for meta-analyses involving 10 or more studies. This involved visual inspection of funnel plots and formal statistical testing using Egger's regression test. A two-sided p-value < 0.10 for Egger's test was considered indicative of potential funnel plot asymmetry. For all other analyses, a two-sided p-value < 0.05 was deemed statistically significant.

Results

Of the 13 studies analyzed, the overall risk of bias is judged to be low, with 12 exhibiting low risk across all domains due to robust randomization, blinding, low attrition, objective outcome measurement, and pre-specified analysis plans. Li et al. (2023) [11] raised some concerns overall due to potential bias from uneven dropout rates between treatment groups.

Data Analysis

Percentage change in bodyweight from baseline: Analysis of 12 RCTs demonstrated that treatment with GLP-1 RAs resulted in a statistically significant reduction in mean percentage body weight compared to control, with an overall MD of -12.79% (95% CI: -15.12 to -10.46, P < 0.0001). Notwithstanding substantial heterogeneity (I² = 97.3%), the overall finding proved robust in a leave-one-out sensitivity analysis. Subgroup analysis revealed significant differences between drug classes (P < 0.0001). Semaglutide treatment (seven trials) resulted in an MD of -11.37% (95% CI: -12.73 to -10.02; I² = 95.1%), while tirzepatide (four trials) showed a greater reduction with an MD of -17.03% (95% CI: -20.27 to -13.78; I² = 88.2%). Noiiglutide (one trial) demonstrated a more modest effect with an MD of -5.44% (95% CI: -7.06 to -3.82).

Participants with 10% or more bodyweight reduction: Analysis of 11 RCTs, encompassing 7,433 participants (4,973 in the intervention arms and 2,460 in control arms), demonstrated that treatment with GLP-1 RAs significantly increased the likelihood of achieving a ≥10% reduction in body weight compared to control conditions (RR = 5.56, 95% CI: 4.25 to 7.26, P < 0.0001). Notwithstanding substantial heterogeneity (I² = 79.8%), the overall finding proved robust in a leave-one-out sensitivity analysis. Subgroup analyses confirmed the efficacy of both semaglutide (six trials, n = 3,629; RR = 5.15, 95% CI: 3.66-7.23) and tirzepatide (four trials, n = 3,550; RR = 6.90, 95% CI: 3.66-13.02); however, the test for subgroup differences indicated that the effect size between these two agents was not statistically significant (P = 0.6878). The Egger's test result (p-value = 0.087) indicates that there is statistically significant evidence of funnel plot asymmetry.

Participants with 15% or more bodyweight reduction: Analysis of 10 RCTs, encompassing 7,179 participants (4,781 in the intervention arms and 2,398 in control arms), demonstrated that treatment with GLP-1 RAs profoundly increased the likelihood of achieving a ≥15% reduction in body weight compared to control conditions (RR = 9.50, 95% CI: 6.68 to 13.51, P < 0.0001). Notwithstanding substantial heterogeneity (I² = 69.0%), the overall finding proved robust in a leave-one-out sensitivity analysis. Subgroup analyses confirmed the high efficacy of both semaglutide (six trials, n = 3,629; RR = 7.29, 95% CI: 5.26-10.11) and tirzepatide (four trials, n = 3,550; RR = 12.68, 95% CI: 6.14-26.18). The test for subgroup differences was not statistically significant (P = 0.2377). The Egger's test result (p-value = 0.0625) indicates that there is statistically significant evidence of funnel plot asymmetry.

Participants with 20% or more bodyweight reduction: Analysis of nine RCTs, encompassing 6,969 participants (4,640 in the intervention arms and 2,329 in control arms), demonstrated that treatment with GLP-1 RAs dramatically increased the likelihood of achieving a ≥20% reduction in body weight compared to control conditions (RR = 15.00, 95% CI: 11.56 to 19.46, P < 0.0001). In contrast to the other endpoints, no heterogeneity was detected for this outcome (I² = 0.0%). The overall finding proved robust in a leave-one-out sensitivity analysis. Subgroup analyses confirmed the significant efficacy of both semaglutide (six trials, n = 3,629; RR = 13.75, 95% CI: 9.61-19.69) and tirzepatide (three trials, n = 3,340; RR = 16.52, 95% CI: 11.31-24.11). The test for subgroup differences was not statistically significant (P = 0.4916).

Change in BMI: Analysis of eight RCTs demonstrated that treatment with GLP-1 RAs resulted in a statistically significant reduction in BMI compared to control, with an overall MD of -4.80 kg/m² (95% CI: -6.24 to -3.36, P < 0.0001). Notwithstanding substantial heterogeneity (I² = 98.1%), the overall finding proved robust in a leave-one-out sensitivity analysis. Subgroup analysis revealed significant differences between drug classes (P < 0.0001 for subgroup differences). Semaglutide treatment (five trials) resulted in an MD of -4.40 kg/m² (95% CI: -4.77 to -4.02; I² = 66.4%), while tirzepatide (two trials) showed a greater reduction with an MD of -7.55 kg/m² (95% CI: -10.22 to -4.89; I² = 97.0%). Noiiglutide (one trial) demonstrated a more modest effect with an MD of -1.78 kg/m² (95% CI: -2.29 to -1.27).

Change in waist circumference: Analysis of 13 RCTs demonstrated that treatment with GLP-1 RAs resulted in a statistically significant reduction in waist circumference compared to control, with an overall MD of -9.78 cm (95% CI: -11.47 to -8.09, P < 0.0001). Notwithstanding substantial heterogeneity (I² = 95.2%), the overall finding proved robust in a leave-one-out sensitivity analysis. Subgroup analysis revealed that semaglutide treatment (eight trials) resulted in an MD of -8.63 cm (95% CI: -9.74 to -7.52; I² = 90.0%), while tirzepatide (four trials) showed a greater reduction with an MD of -12.83 cm (95% CI: -14.72 to -10.94; I² = 61.6%). Noiiglutide (one trial) demonstrated a more modest effect with an MD of -3.89 cm (95% CI: -5.59 to -2.19). The test for subgroup differences was statistically significant (P < 0.0001).

Change in systolic blood pressure: Analysis of 12 RCTs demonstrated that treatment with GLP-1 RAs resulted in a statistically significant reduction in systolic blood pressure compared to control, with an overall MD of -6.32 mmHg (95% CI: -7.92 to -4.72, P < 0.0001). Notwithstanding substantial heterogeneity (I² = 87.8%), the overall finding proved robust in a leave-one-out sensitivity analysis. Subgroup analysis showed no statistically significant differences between drug classes (P = 0.0879 for subgroup differences). Semaglutide treatment (eight trials) resulted in an MD of -5.15 mmHg (95% CI: -6.40 to -3.90; I² = 75.4%), while tirzepatide (three trials) showed a greater reduction with an MD of -9.45 mmHg (95% CI: -13.38 to -5.51; I² = 83.8%). Noiiglutide (one trial) demonstrated a reduction of -4.32 mmHg (95% CI: -6.91 to -1.73). ). The Egger's test result (p-value = 0.0048) indicates that there is statistically significant evidence of funnel plot asymmetry.

Change in diastolic blood pressure: Analysis of 11 RCTs demonstrated that treatment with GLP-1 RAs resulted in a statistically significant reduction in diastolic blood pressure compared to control, with an overall MD of -1.95 mmHg (95% CI: -3.21 to -0.69, P = 0.0024). Notwithstanding substantial heterogeneity (I² = 90.9%), the overall finding proved robust in a leave-one-out sensitivity analysis. Subgroup analysis revealed no statistically significant differences between drug classes (P = 0.6060 for subgroup differences). Semaglutide treatment (seven trials) resulted in an MD of -2.12 mmHg (95% CI: -3.29 to -0.94; I² = 82.5%), while tirzepatide (three trials) showed an MD of -2.90 mmHg (95% CI: -7.82 to 2.02; I² = 96.6%) that was not statistically significant (P = 0.2507). Noiiglutide (one trial) demonstrated a reduction of -0.99 mmHg (95% CI: -2.82 to 0.84). The Egger's test result (p-value = 0.1185) indicates that there is no statistically significant evidence of funnel plot asymmetry.

Safety Profile

**Figure 3:**
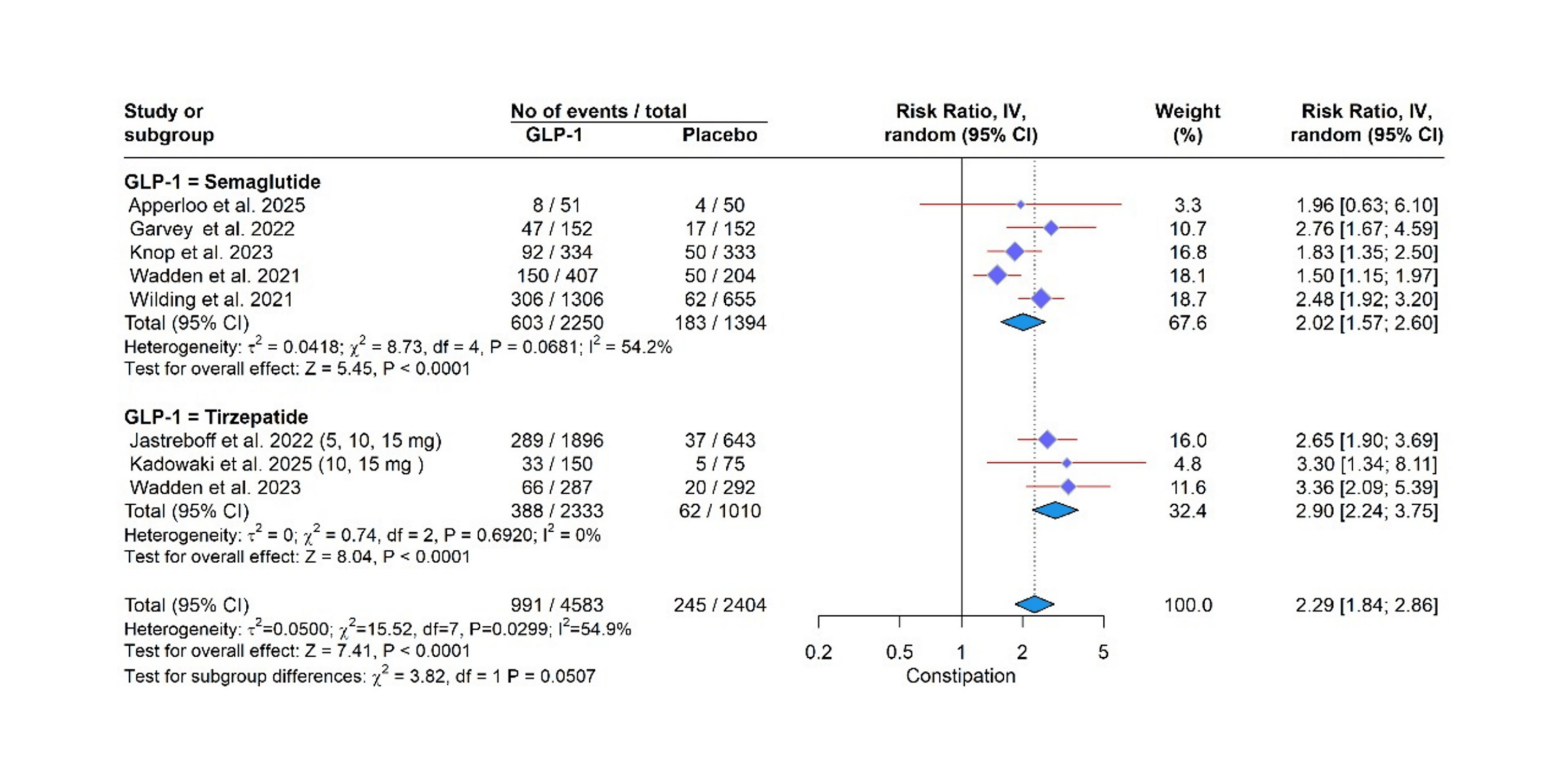
Constipation. GLP-1: glucagon-like peptide-1. Apperloo et al. (2025) [5], Garvey et al. (2022) [7], Jastreboff et al. (2022) [8], Kadowaki et al. (2025) [9], Knop et al. (2023) [10], Wadden et al. (2021) [14], Wadden et al. (2023) [15], Wilding et al. (2021) [16].

**Figure 4:**
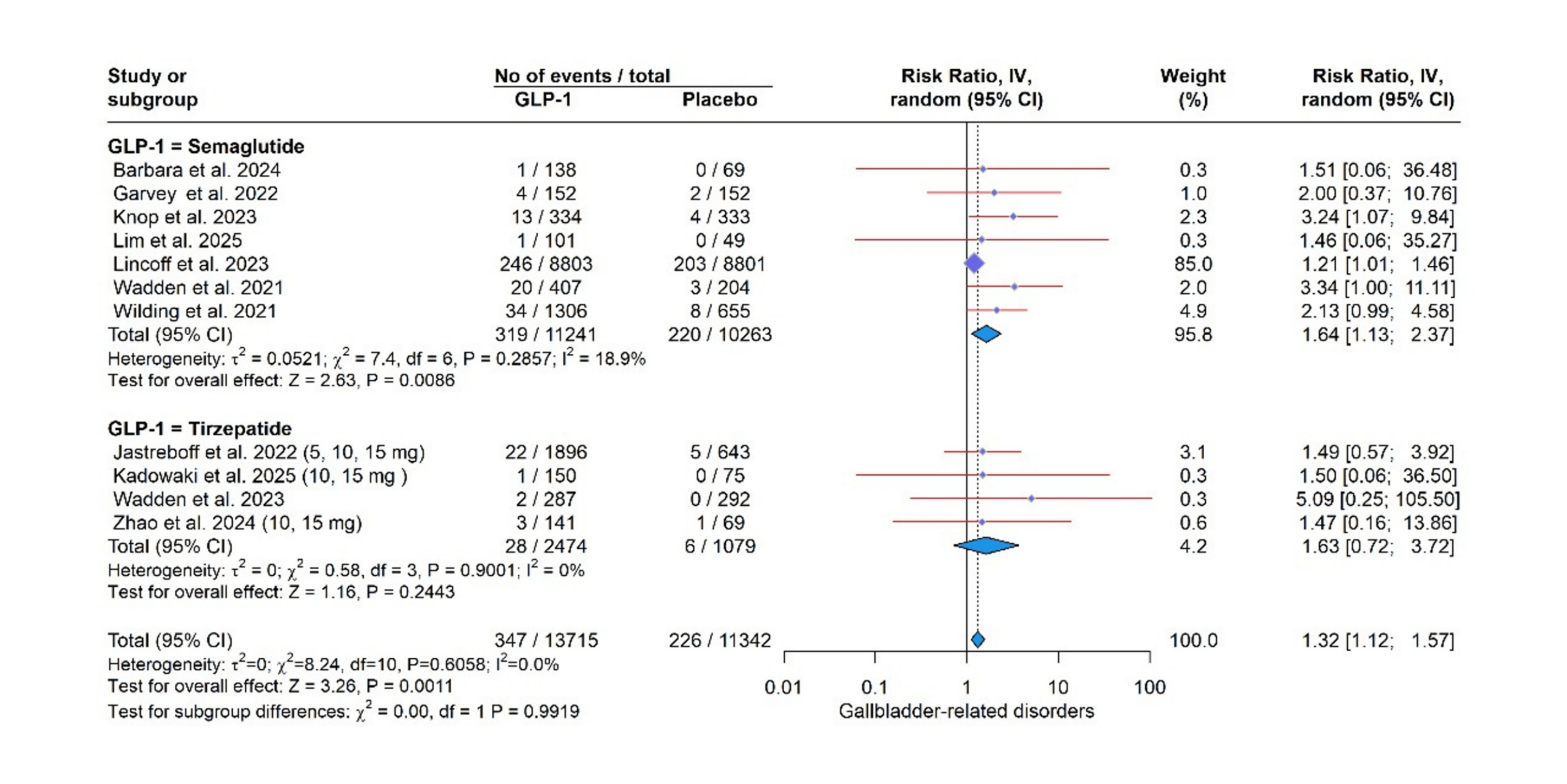
Gallbladder-related disorders. GLP-1: glucagon-like peptide-1. Barbara et al. (2024) [6], Garvey et al. (2022) [7], Jastreboff et al. (2022) [8], Kadowaki et al. (2025) [9], Knop et al. (2023) [10], Lim et al. (2025) [12], Lincoff et al. (2023) [13], Wadden et al. (2021) [14], Wadden et al. (2023) [15], Wilding et al. (2021) [16], Zhao et al. (2024) [17].

**Figure 5:**
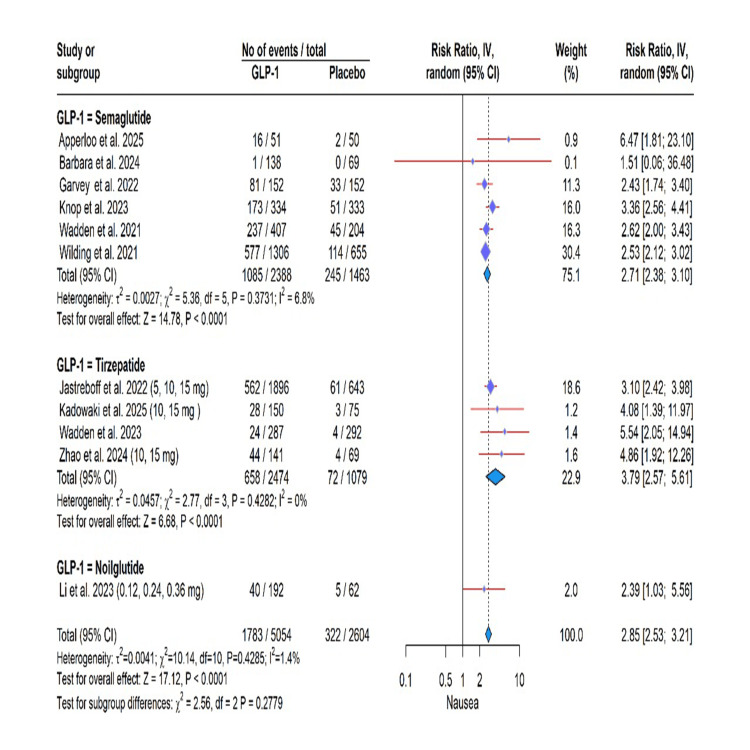
Nausea symptoms. GLP-1: glucagon-like peptide-1. Apperloo et al. (2025) [5], Barbara et al. (2024) [6], Garvey et al. (2022) [7], Jastreboff et al. (2022) [8], Kadowaki et al. (2025) [9], Knop et al. (2023) [10], Wadden et al. (2021) [14], Wadden et al. (2023) [15], Wilding et al. (2021) [16], Zhao et al. (2024) [17].

**Figure 6:**
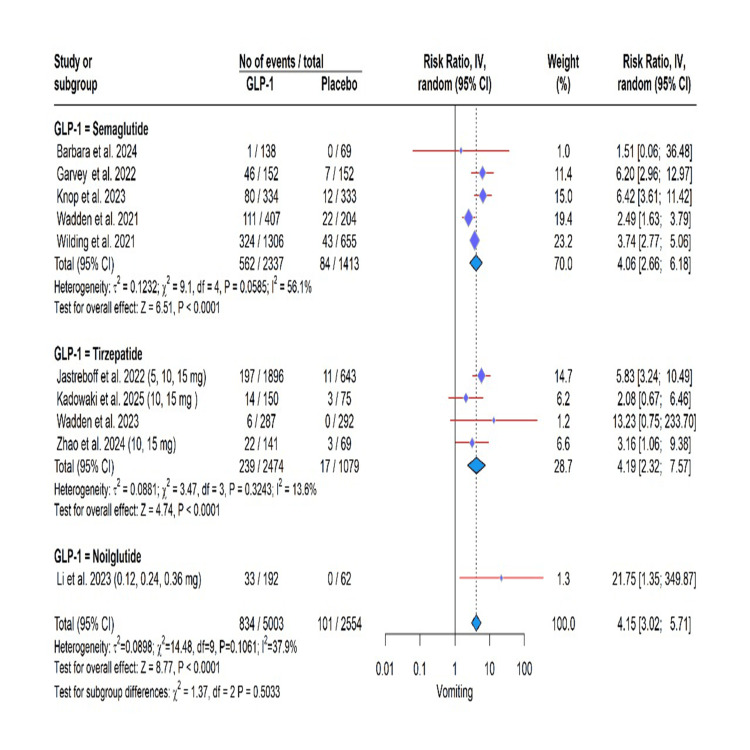
Vomiting data. GLP-1: glucagon-like peptide-1. Barbara et al. (2024) [6], Garvey et al. (2022) [7], Jastreboff et al. (2022) [8], Kadowaki et al. (2025) [9], Knop et al. (2023) [10], Wadden et al. (2021) [14], Wadden et al. (2023) [15], Wilding et al. (2021) [16], Zhao et al. (2024) [17].

**Figure 7:**
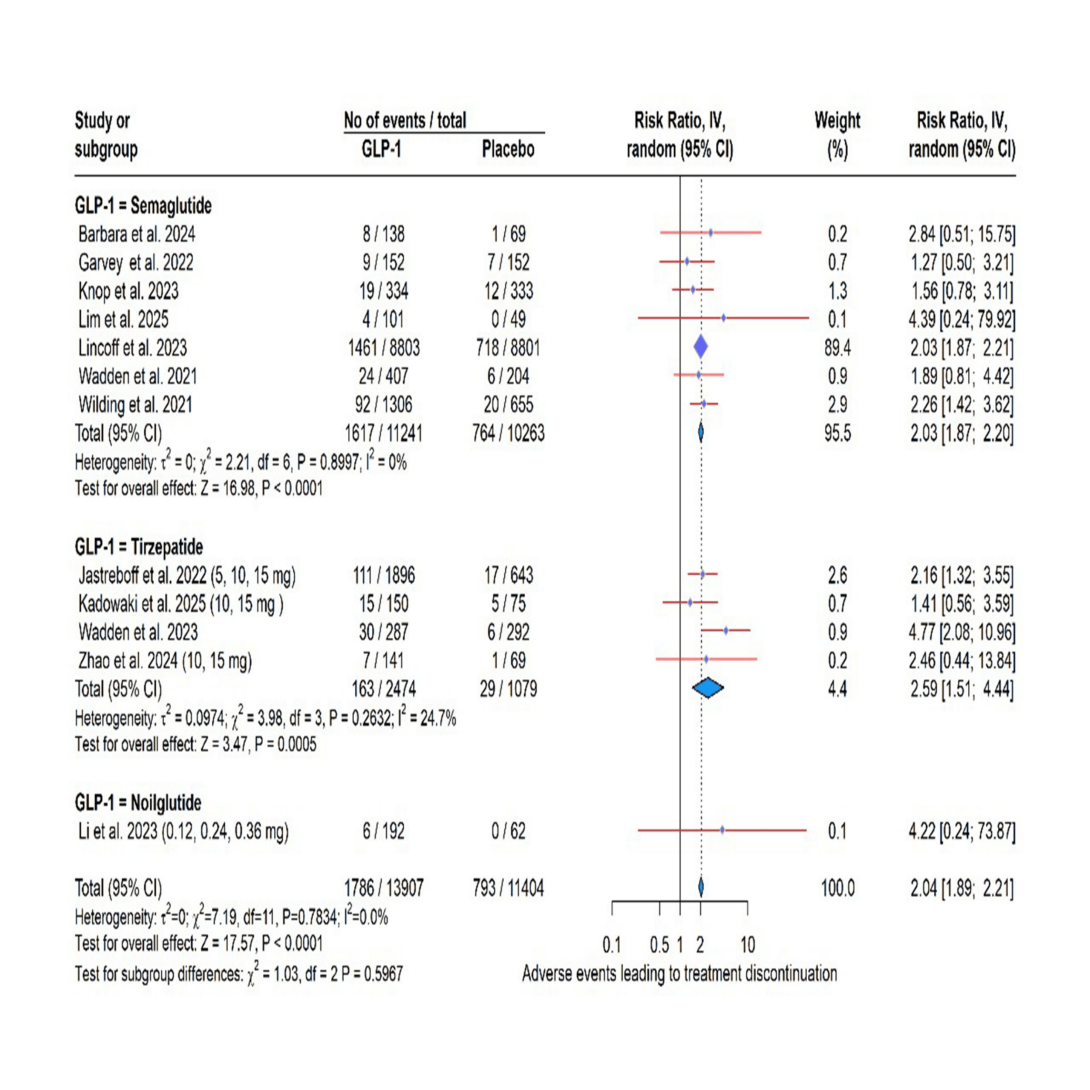
Adverse event leading to treatment discontinuation. GLP-1: glucagon-like peptide-1. Barbara et al. (2024) [6], Garvey et al. (2022) [7], Jastreboff et al. (2022) [8], Kadowaki et al. (2025) [9], Knop et al. (2023) [10], Lim et al. (2025) [12], Lincoff et al. (2023) [13], Wadden et al. (2021) [14], Wadden et al. (2023) [15], Wilding et al. (2021) [16], Zhao et al. (2024) [17].

GLP-1 RA use was significantly associated with an elevated risk of several adverse events in this meta-analysis. Specifically, the risk was most pronounced for gastrointestinal events, including vomiting (RR: 4.15, 95% CI: 3.02 to 5.71, P < 0.0001; I² = 37.9%), nausea (RR: 2.85, 95% CI: 2.53 to 3.21, P < 0.0001; I² = 1.4%), diarrhea (RR: 2.85, 95% CI: 2.53 to 3.21, P < 0.0001; I² = 1.4%), constipation (RR: 2.29, 95% CI: 1.84; 2.86, P < 0.0001; I² = 54.9%), and decreased appetite (RR: 2.65, 95% CI: 2.07 to 3.40, P < 0.0001; I² = 0.0%). A significantly higher risk was also observed for gallbladder-related disorders (RR: 1.32, 95% CI: 1.12 to 1.57, P = 0.0011; I² = 0.0%) and adverse events leading to treatment discontinuation (RR: 2.04, 95% CI: 1.89 to 2.21, P < 0.0001; I² = 0.0%). In contrast, a protective effect was found for cardiovascular disorders (RR: 0.77, 95% CI: 0.64, 0.93, P = 0.0074; I² = 0.0%). Importantly, the incidence of serious adverse effects was not significantly different from placebo (RR: 1.08, 95% CI: 0.89 to 1.32, P = 0.43; I² = 41.7%). The test for subgroup differences was not significant for any outcome (all P > 0.05), indicating a consistent effect across semaglutide, tirzepatide, and noiiglutide (Figures 3-7).

Discussion

This systematic review and meta-analysis evaluated the impact of GLP-1 RAs on anthropometric and metabolic outcomes, including body weight, BMI, waist circumference, and blood pressure. The pooled analysis demonstrated that GLP-1 RAs significantly reduce body weight, with an overall mean percentage change of -12.79%. Among individual agents, tirzepatide achieved the greatest reduction (-17.03%), followed by semaglutide (-11.37%) and noiiglutide (-5.44%).

In addition to continuous weight reduction outcomes, GLP-1 RAs markedly increased the probability of achieving clinically relevant thresholds of ≥5%, ≥10%, ≥15%, and ≥20% body weight loss. For example, the relative risk of reaching ≥20% weight loss was 15.00, highlighting the strong efficacy of these agents. Both tirzepatide and semaglutide were highly effective across these benchmarks, and although tirzepatide yielded a greater mean reduction, differences between the two were not statistically significant when categorical weight loss outcomes were compared.

Other metabolic parameters also improved significantly. Pooled results showed decreases in BMI (MD: -4.80 kg/m²), waist circumference (-9.78 cm), systolic blood pressure (-6.32 mmHg), and diastolic blood pressure (-1.95 mmHg). Tirzepatide again demonstrated more pronounced effects on BMI and waist circumference compared to semaglutide and noiiglutide, while reductions in blood pressure were consistent across the drug class.

These findings reinforce the expanding body of evidence supporting GLP-1 RAs as effective agents for weight management and cardiometabolic health. Prior meta-analyses have documented similar benefits in individuals with overweight or obesity [4]. For instance, a study by Tanashat et al. (2025) [18] reported average weight loss of -8.53 kg, alongside favorable effects on lipid levels, blood pressure, and a reduced risk of myocardial infarction [18]. The observed mean body weight reduction of -12.79% in this study exceeds results from several earlier reviews, which typically reported losses in the 5-10% range [19]. A study by Karagiannis et al. (2024) [20] also demonstrated that tirzepatide produced greater weight reductions across all tested doses compared with semaglutide, with approximately 9.57 kg lost at higher tirzepatide doses versus 4.97 kg with semaglutide 2.0 mg [20]. The superior weight loss associated with tirzepatide in our analysis is consistent with head-to-head trials and meta-analyses, indicating that dual glucose-dependent insulinotropic polypeptide (GIP) and GLP-1 receptor agonism contributes to its enhanced efficacy [21].

The increased likelihood of achieving incremental thresholds of weight loss (≥5%, ≥10%, ≥15%, and ≥20%) underscores the clinical importance of GLP-1 RAs. Weight reductions of ≥5% are generally considered sufficient to improve obesity-related comorbidities, while losses of ≥10-15% confer even greater health benefits [14]. Our findings, particularly the high rates of participants attaining ≥15% and ≥20% reductions, highlight the transformative role of these therapies in obesity care. These outcomes are in line with the results of large-scale RCTs by Wilding et al. [16] and Jastreboff et al. [8], both of which reported high proportions of patients reaching these thresholds with semaglutide and tirzepatide.

A study by Després et al. (2006) [22] emphasized improvements in BMI, waist circumference, and blood pressure observed reflect the pleiotropic actions of GLP-1 RAs beyond glucose regulation. Reductions in abdominal adiposity are particularly important given the close relationship between central obesity, metabolic syndrome, and cardiovascular risk [13]. Similarly, a study by Marso et al. (2016) [23] demonstrated cardiovascular benefits of GLP-1 RAs, consistent with the blood pressure reductions reported in our analysis. Although the absolute changes in diastolic blood pressure were modest, the consistent reductions across the class support their broader cardioprotective effects.

Limitations and future directions

Despite strong evidence, several limitations should be acknowledged. First, substantial heterogeneity was detected in outcomes such as body weight, BMI, and waist circumference. Sensitivity analyses supported the overall robustness of results, yet high I² values point to variability among trials, likely due to differences in study populations, durations, interventions, and dosages. Further exploration of these factors is warranted.

Second, evidence for noiiglutide was limited, with only one trial available for most endpoints. This restricts the ability to draw firm conclusions regarding its relative efficacy and safety compared to semaglutide and tirzepatide. Additional high-quality RCTs are needed to establish its role in obesity management. Third, although reductions in blood pressure were statistically significant, the clinical relevance of changes in diastolic blood pressure may be limited. Long-term outcome studies are necessary to determine whether these reductions translate into meaningful cardiovascular benefits.

We acknowledge that the trials had varying endpoints, with most having follow-up periods exceeding 52 weeks. This variability in endpoints may impact our conclusions.

Future research priorities include long-term evaluations of the durability of weight loss and prevention of weight regain, as well as studies in diverse patient subgroups defined by comorbidities or ethnicity. Economic analyses of GLP-1 RA use, alongside integration with lifestyle and behavioral interventions, will be crucial for guiding clinical practice. Moreover, direct comparative trials between GLP-1 RAs and novel combination regimens will provide insights into optimal therapeutic strategies.

## Conclusions

This systematic review and meta-analysis provide robust evidence that GLP-1 RAs are highly effective for producing clinically significant weight loss and improving multiple cardiometabolic outcomes in individuals with overweight or obesity. While benefits are seen across all agents, tirzepatide appears to provide the most substantial weight reduction relative to semaglutide. Collectively, these findings highlight the critical role of GLP-1 RAs as cornerstone therapies in the comprehensive treatment of obesity and its related complications.
